# Persistent poverty and late-stage breast cancer diagnosis in the United States: Impacts of rural residence, race, and time within Surveillance Epidemiology and End Results registries, 2004 to 2021

**DOI:** 10.21203/rs.3.rs-6933779/v1

**Published:** 2025-06-27

**Authors:** Heather R. Sherr, Amr S. Soliman, Kimberly A. Bertrand, Kelly A. Hirko

**Affiliations:** Boston University School of Public Health; City University of New York Medical School; Slone Epidemiology Center at Boston University; Michigan State University

**Keywords:** Persistent poverty, rural/urban status, race, late-stage breast cancer

## Abstract

**Background:**

County-level poverty is associated with higher rates of late-stage breast cancer (LSBC). The role of persistent poverty (> 20% residents in poverty for 30 + years) in breast cancer stage at diagnosis is unclear and may vary by rural/urban status, race, and over time.

**Methods:**

We analyzed county-level data from the Surveillance, Epidemiology, and End Results Program for females (ages 20–74 years) with late-stage (regional and distant) breast cancer from 2017–2021 (excluding 2020). We estimated mean rate differences (RDs) in LSBC between persistently poor (n = 156) and non-persistently poor (n = 923) counties using multivariable linear regression models stratified by rural/urban status and race. We used Joinpoint regression analysis to estimate overall annual percent changes (APC) in breast cancer rates by stage from 2004–2019, as well as by persistent poverty and rural/urban status.

**Results:**

Age-adjusted LSBC rates were higher in persistently poor counties, but differences were diminished in multivariable models (RD = 2.74, 95% CI=-1.6, 7.1). Adjusted results did not differ based on rural/urban status (p_int_=0.94) or race (p_int_=0.08). LSBC rates declined from 2004–2017 but increased from 2017–2019, particularly in persistently poor counties (APC = 5.54, 95% CI = 0.06, 8.69). LSBC rates declined from 2004–2019 in urban (APC =−0.87, 95% CI=−1.63, −0.65) but not rural (APC=−0.01, 95% CI =−0.34, 0.32) counties.

**Conclusion:**

Elevated LSBC rates in persistent poverty counties are largely explained by recent poverty and race/ethnicity. Given the rising rates of LSBC in persistent poverty counties, our findings emphasize the importance of addressing breast cancer screening barriers among disadvantaged populations.

## Introduction

One in eight women in the United States are diagnosed with breast cancer over their lifetime, making breast cancer the most common cancer among U.S. women. Breast cancer incidence has increased in recent decades; this increase has been driven by early-stage diagnosis [1–3]. Early detection, facilitated by widespread mammography screening, is a critical determinant of survival outcomes, with 5-year relative survival rates for localized, regional, and distant cases being 99%, 87%, and 32%, respectively [3]. Late-stage diagnoses, defined as regional and distant stage disease (stages II, III, and IV), have remained stable over time and account for more than one third of all breast cancer diagnoses in the United States [4].

Prior findings suggest socioeconomic disparities in breast cancer screening utilization, stage at diagnosis, and survival. For example, individuals with lower socioeconomic status are less likely to be screened for breast cancer and are more likely to be diagnosed at advanced stages of disease [5]. U.S. counties with higher poverty rates show increased incidence of LSBC compared to those with lower poverty rates [6]. Additionally, poverty has also been linked to breast cancer survival, with lower survival rates evident in high-poverty U.S. counties and among women with lower household incomes [7, 8].

Persistent poverty is a measure of sustained, community-level poverty. Counties or census tracts where 20% or more of the population has lived below the poverty level for approximately 30 years are considered persistently poor. In a recent analysis of U.S. population-based data, residing in persistently poor counties was associated with a 10% higher risk of breast cancer-specific mortality [9]. Individuals residing in these areas may experience barriers to breast cancer survival, including delayed screening and treatment, regardless of their own poverty status [10]. Persistent poverty may also promote chronic stress, weakening the immune system, inhibiting apoptosis and potentially facilitating rapid progression to regional or distant stages [11, 12]. Although higher odds of LSBC diagnosis have been documented in persistently poor counties [13], prior studies did not adjust for current poverty, making it di cult to discern whether long-term and immediate socioeconomic disadvantage have distinct effects on breast cancer stage at diagnosis. Thus, the role persistent poverty in breast cancer stage at diagnosis, independent of recent poverty, remains unclear.

Associations between persistent poverty and LSBC may also be modified by rural/urban status and race, which are associated with breast cancer screening utilization [5, 14]. Both rural/urban status and Non-Hispanic Black race have been associated with higher rates of LSBC incidence and breast cancer mortality [6, 15–17]. While previous studies observed positive associations between persistent poverty and LSBC after adjusting for rural/urban status, race, insurance status, and education [13], these findings do not consider the moderating roles of rural/urban status and race. Disparities in LSBC risk may be due to the combined effects of poverty, race, and rural/urban status, and understanding the varying roles of these factors relative to persistent poverty and over time will be paramount to developing and implementing effective interventions to address disparities.

The purpose of this study was to examine associations between county-level persistent poverty and LSBC rates accounting for recent poverty levels and to evaluate the potential modulating role of rural/urban status and race. Considering the overall increase in early-stage breast cancer rates and the potential impact of the COVID-19 pandemic on breast cancer stage at diagnosis, we also assessed trends in breast cancer rates by stage at diagnosis over time and according to county-level persistent poverty and rural/urban status [18–20].

## Methods

### Study Population

We obtained data on county-level age-adjusted breast cancer incidence rates by stage at diagnosis (i.e., localized, regional, distant) through the National Cancer Institute’s Surveillance, Epidemiology, and End Results (SEER) Research Plus Limited-Field 22 database. The SEER Program is a comprehensive source of population-based information on cancer diagnoses, treatment, and survival collected on approximately 48% of the United States population; this data is considered generalizable to the entire United States [21]. The selected database, released in April of 2024, contained diagnostic information from 22 cancer registries across 16 states between 2004 and 2021.

For the primary analysis, our study population included 1,079 counties with age-adjusted breast cancer incidence rates by stage during the years 2017–2019 and 2021. The year 2020 was excluded from our analysis due to concerns with data quality [19]. We restricted our study population to females between the ages of 20 and 74 years at diagnosis, as breast cancer screening recommendations include women up to age 74 [22]. We excluded the following counties with missing information on persistent poverty; the Alaska Native Registry, Clark County, Idaho, Borden County, Texas, Glasscock County, Texas, Kenedy County, Texas, King County, Texas, and Loving County, Texas). In our secondary analysis of breast cancer stage of diagnosis trends over time, we included breast cancer cases from 2004 through 2019 within the same subset of U.S. counties.

### Primary Exposure

The primary exposure of interest in this analysis was persistent poverty, which identifies counties that have experienced high rates of poverty for extended periods [10]. It is based on poverty data from the 1990 and 2000 decennial censuses, as well as the 2007–2011 and 2015–2019 American Community Survey (ACS) 5-year estimates. Persistent poverty is a two-level indicator; counties are considered persistently poor if 20% or more of the population has lived below the poverty line for a period of about 30 years.

### Primary Outcome

The primary outcome of our analysis was county-level age-adjusted LSBC incidence rate per 100,000 person-years. We defined breast cancer cases using the ICD-O-3/WHO 2008 site recode [23]. Stage at diagnosis was determined using the summary/historical combined summary stage (2004+) metric available in SEER. Following previous studies, we defined LSBC as regional (stage II) or distant (stage III and IV) diagnosis [6, 24]. For the Joinpoint analysis, we ascertained data for total breast cancer rates, as well as for early (localized, stage I) and late-stage (regional and distant, stage II, III, and IV) breast cancer diagnoses between 2004 and 2021. Building off a prior study assessing the impact of COVID-19 pandemic on breast cancer stage at diagnosis [19], we examined breast cancer rates within multiple time periods: overall (2017–2021, excluding 2020) before the COVID-19 pandemic (2017–2019) and during the pandemic (2020 and 2021).

### Covariates and Stratifying Variables

We obtained county-level data on the following potential covariates based on prior knowledge and conceptualization of directed acyclic graphs (DAGs): county-level recent poverty (defined as county-level percentages of individuals living below the poverty line since 2017), rural-urban continuum code, % without health insurance, % unemployed, % Non-Hispanic Black, % Hispanic or Latino, and % with a bachelor’s degree or higher. All county-level covariate data was collected from ACS 5-year estimates between 2017 and 2021 [25]. The DAG that informed our selection of covariates is shown in Supplemental Fig. 1.

We assessed county-level rural/urban status and the percentage of Non-Hispanic Black individuals in each county as stratifying variables. Rural/urban status was defined using the 2013 rural urban continuum codes (RUCC) developed by the United States Department of Agriculture (USDA), which were included in the SEER program [26]. RUCC codes divide countries into nine categories. Counties with a code of 1–3 are considered metropolitan, or urban, while counties with a score of 4–9 are considered nonmetropolitan, or rural. We were unable to stratify our analysis by U.S. census region due to small numbers of persistent poverty counties in the Midwest (n = 3) and Northeast (n = 1). Since the county-level percentage of Non-Hispanic Black individuals was associated with both persistent poverty and LSBC diagnosis in our data, we also stratified our results by the county % of Non-Hispanic Black residents. After visually inspecting the distribution of Non-Hispanic Black individuals across counties, we created a dichotomous variable of counties with a Non-Hispanic Black population of ≤ 5% or > 5% for strati cation. We also included more specific measures of these dichotomous variables (rural-urban continuum code and % Non-Hispanic Black) to capture within-stratum variations in the stratified models.

### Statistical Analysis

We accessed county-level age-adjusted LSBC incidence rates between 2004 and 2021, 2013 RUCC codes, and 2015–2019 persistent poverty status using the SEER*Stat program [27]. We merged this data with ACS 5-year estimates in Excel by state and county name. We conducted descriptive analysis for all counties, examining differences in county characteristics by persistent poverty status. We used t-tests to compare the mean LSBC rates by persistent poverty status, stratifying by rural/urban status and race. Additionally, linear correlations between persistent and recent poverty, as well as persistent poverty and percentage Non-Hispanic Black residents were assessed by generating Pearson correlation coefficients and conducting simple linear regression analysis using these metrics as exposure variables.

Linear regression models were created to examine the association between county-level persistent poverty and LSBC incidence rates. First, we assessed an age-adjusted model including data from the years 2017–2021, excluding the year 2020. Multivariable models adjusted for county-level recent poverty, rural-urban continuum code, % without health insurance, % unemployed, % Non-Hispanic Black, % Hispanic or Latino, and % with a bachelor’s degree or higher. The percentage of females who received a mammogram in the previous 2 years was not included in multivariable models, as this variable showed collinearity with the intercept term in the model. We concluded that receiving a mammogram demonstrates the complex interaction of socio-economic and geographical barriers included in the model and, therefore, proved to be redundant. The inclusion of this variable in our multivariable models did not change our interpretation of subsequent results.

Results are reported as linear estimates of the rate difference (RD) between persistent poverty and nonpersistent poverty counties. Precision of RD estimates were assessed using 95% confidence intervals. The significance of interactions with rural/urban status and race was determined by using interaction terms in the multivariable model. To further explore the moderation of association between persistent poverty and LSBC rates by race, we conducted secondary analysis of the 592 counties in the South census region of the United States.

We used SEER’s Joinpoint regression program, which creates log-transformed linear regression plots, to examine trends in breast cancer stage at diagnosis rates over time [28]. We calculated annual percent change (APC) and 95% confidence intervals between each inflection or deflection point for the years 2004–2019. Though diagnosis rates were plotted for the years 2020 and 2021, these years were not included in APC calculations due to previously discussed data quality concerns. This study was conducted with the approval of the Michigan State University Institutional Review Board (IRB) for Human Subjects Research. All analysis was conducted with SAS version 9.4 software [29]. P-values < 0.05 were considered statistically significant.

## Results

Of the 1,079 counties in the study, 156 (14.4%) were persistently poor and 639 (59.2%) were rural (Table 1). Among persistently poor counties, 127 (81.4%) were rural, compared to 55.5% in non-persistently poor counties. Persistent poverty counties were primarily located in the South (88.5%) and had a higher proportion of Non-Hispanic Black residents, recent poverty, unemployment, lack of health insurance, and lower educational attainment compared to non-persistent poverty counties.

As shown in Table 2, persistent poverty counties showed elevated rates of LSBC compared to non-persistent poverty counties (58.98 vs. 53.20/100,000 person-years; p = 0.005). This disparity in LSBC diagnosis was more pronounced in rural counties (58.61 vs. 52.20/100,000 person-years; p = 0.005) and in counties with higher Non-Hispanic Black populations (61.67 vs. 55.4/100,000 person-years; p = 0.03). Differences in LSBC rates by persistent poverty status were not significant in urban counties (p = 0.12) or in those with a lower proportion of Non-Hispanic Black residents (p = 0.13).

Age-adjusted LSBC rates were higher in persistently poor counties, but the difference was attenuated in multivariable models (RD = 2.74, 95% CI=−1.6, 7.1) (Table 3). Persistent poverty was positively correlated with both recent poverty (ρ = 0.62) and % Non-Hispanic Black (ρ = 0.35) but did not demonstrate collinearity with either variable. Additionally, simple linear regression analysis with recent poverty or % Non-Hispanic Black residents as exposure variables showed slight association with the outcome (RD Non-Hispanic Black = 0.29, 95% CI = 0.20, 0.37, RD recent poverty = 0.27, 95% CI = 0.08, 0.44), but not to the degree of persistent poverty (RD = 5.78, 95% CI = 2.5, 9.1).

In stratified analysis, persistently poor counties had higher rates of LSBC diagnosis in both rural (RD = 6.41, 95% CI = 2.0, 10.9) and urban settings (RD = 6.16, 95% CI = 1.1, 11.2), though RDs were similarly attenuated in the adjusted models. The association of persistent poverty and LSBC rates did not differ according to rural/urban status (p_int_=0.94). In age-adjusted models, LSBC rates were higher in persistently poor counties with > 5% Non-Hispanic Black residents only (RD = 6.23, 95% CI = 2.12, 10.34). However, in the multivariable model, higher LSBC rates were observed in persistently poor counties with a smaller proportion of Non-Hispanic Black individuals (RD = 7.23, 95% CI = 0.2, 14.2) with non-significant interaction by race (p_int_=0.08).

Results from secondary analysis of the 592 counties in the South census region of the United States are presented in Supplemental Table 1. In this analysis, LSBC rates were higher in persistent poverty than nonpersistent poverty counties, overall (59.72 vs. 54.63/100,000 person-years; p = 0.03) and within rural counties (59.09 vs. 53.11/100,000 person-years; p = .03) and counties with > 5% Non-Hispanic Black residents (61.74 vs. 56.48/100,000 person-years; p = 0.03). While age-adjusted models show significant differences in LSBC diagnosis rates between persistently poor and non-persistently poor counties in the South (RD = 5.11, 95% CI = 1.00, 9.21), specifically rural counties (RD = 5.99, 95% CI = 0.66, 11.31) and those with a Non-Hispanic Black population of < 5% (RD = 5.26, 95% CI = 0.44, 10.08), differences were completely attenuated after adjusting for recent poverty and race.

**Figure 1:**
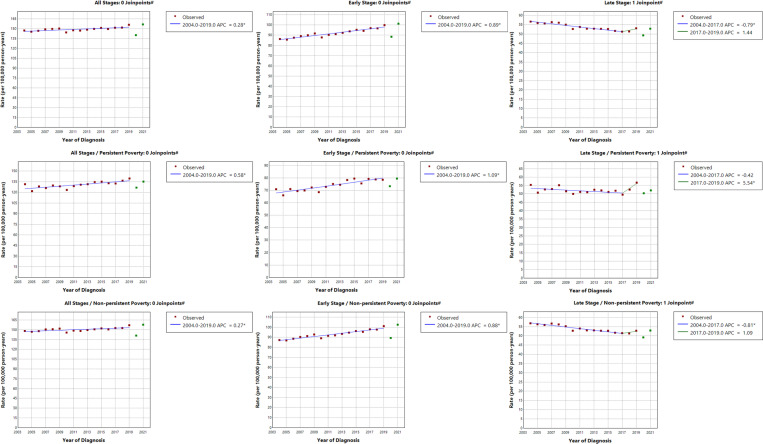
Annual percent changes (APCs) of age-adjusted breast cancer incidence rates (per 100,000 person-years) from 2004–2019 by stage at diagnosis overall and stratified by persistent poverty status. * Annual percent change (APC) is significantly different from zero at the alpha = 0.05 level. ^#^ The years 2020 and 2021 were excluded from model fitting.

**Figure 2:**
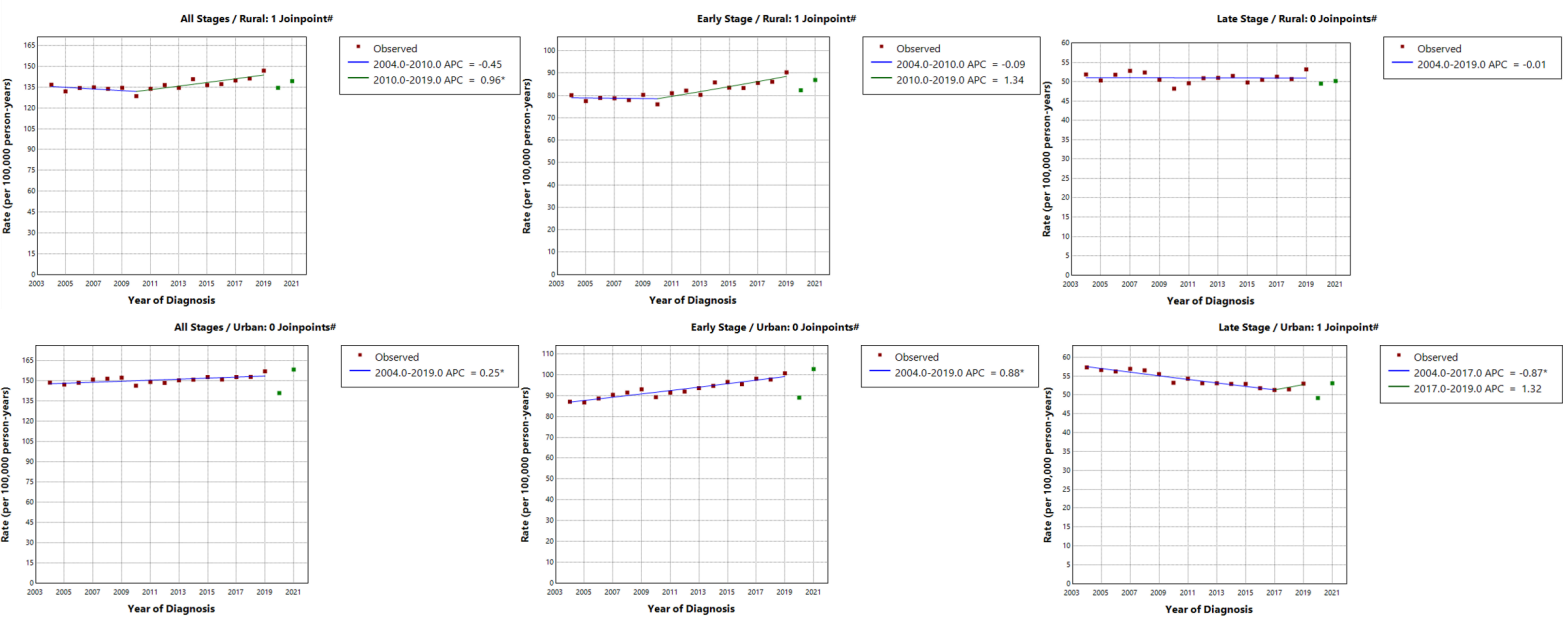
Annual percent changes (APCs) of age-adjusted breast cancer incidence rates (per 100,000 person-years) from 2004–2019 by stage at diagnosis and rural/urban status. * Annual percent change (APC) is significantly different from zero at the alpha = 0.05 level. ^#^The years 2020 and 2021 were excluded from model fitting.

As shown in Fig. 1, early-stage breast cancer rates have been on the rise since 2004 (APC = 0.89, 95% CI = 0.69, 1.10). LSBC rates decreased from 2004 to 2017 (APC=−0.79, 95% CI=−1.58, −0.56) but have begun to increase beginning in 2017 (APC = 1.44, 95% CI=−0.67, 2.66). Increases in overall age-adjusted breast cancer rates over time were more pronounced in persistent poverty vs. non-persistent poverty counties (APC = 0.58, 95% CI = 0.25, 0.91) vs. 0.27, 95% CI = 0.10, 0.44). Similar trends were observed for early-stage breast cancer rates (APC = 1.09, 95% CI = 0.70, 1.49 in persistent poverty vs. 0.88 (0.68, 1.08) in non-persistent poverty counties (Fig. 1). LSBC rates began rising in 2017, especially within counties experiencing persistent poverty (APC = 5.54, 95% CI = 0.06, 8.69). In analysis by rural/urban status, overall and early-stage breast cancer rates increased more rapidly in rural counties (Fig. 2). Interestingly, LSBC rates remained relatively constant in rural counties (APC=−0.01, 95% CI =−0.34, 0.32), while rates declined in urban counties through 2017 (APC=−0.87, 95% CI=−1.63, −0.65) with non-significant increase from 2017–2019 (APC = 1.32, 95% CI= −0.74, 2.52).

## Discussion

Overall, rates of LSBC were higher in persistently poor U.S. counties, but the differences were attenuated after adjusting for current poverty levels and race. These findings suggest that recent socioeconomic position indicators in tandem with racial demographics may be more relevant for late-stage breast cancer incidence than longer-term poverty. Interestingly, LSBC rates were higher in persistent poverty counties with smaller Non-Hispanic Black populations in this study, even after adjustment for current poverty. These results warrant additional research to examine associations between persistent poverty and breast cancer stage at diagnosis by race. Our findings also demonstrate rising rates of LSBC in recent years, particularly in persistently poor counties. Moreover, progress in reducing LSBC rates has been limited to urban counties, with stagnant rates over time in rural regions. Taken together, our findings suggest the need for targeted efforts to address breast cancer screening barriers to rural and impoverished areas of the U.S.

In this study, persistent poverty was associated with higher rates of LSBC, which is consistent with results from a prior study [13]. Findings from our study extend our understanding of the role of recently experienced poverty in the observed differences in LSBC rates by persistent poverty status. Indeed, differences in LSBC rates were diminished after adjusting for current poverty and race. Individual-level data analysis may determine the varying impact of these factors on disparities in breast cancer stage at diagnosis. The impact of rural/urban status on breast cancer stage at diagnosis can vary depending on the specific geographic location, though the rural/urban disparity in breast cancer stage at diagnosis observed in our study is consistent with prior research [15, 30]. Subsequent studies should examine factors such as chronic stress, social isolation, diet and physical activity, consumption of alcohol and tobacco, cancer fatalism, and lack of health prioritization, which may contribute to breast cancer disparities in impoverished and rural communities [31–33].

Persistent poverty counties had higher rates of LSBC than their non-poor counterparts, regardless of the proportion of Non-Hispanic Black residents. However, in multivariable models, differences in LSBC rates in relation to persistent poverty persisted only in counties with smaller Non-Hispanic Black populations. Selected covariates, such as unemployment, uninsurance, and education, may better explain the differences in LSBC rates in counties with larger Black populations, as these factors disproportionately affect Non-Hispanic Black individuals due to systemic racism [34]. Moreover, Non-Hispanic Black women are more likely to be diagnosed with more aggressive breast cancer subtypes, which warrants future analysis of subtype-specific differences according to persistent poverty and race [35].

Our study is the first to our knowledge, to assess trends in LSBC by persistent poverty, rural/urban status, and race and over the years impacted by the COVID-19 pandemic. While we observed an overall increase in early-stage breast cancer and decrease in late-stage disease from 2004–2017, which coincides with the documented rise in early breast cancer detection [1], we also noted an increase in LSBC rates beginning in 2017. It is too early to know if this increase is due to SEER’s updated staging criteria employed in 2018, which placed patients with larger tumors and higher numbers of positive lymph nodes into higher stage categories due to increased risk of recurrence or metastasis [36]. Analysis of trends in 2022 and 2023 is needed to understand the impact of the COVID-19 pandemic on breast cancer diagnosis by stage more fully. Much like in previous studies, analysis of trends in cancer diagnosis can be conducted within state registries [19] to inform the development of targeted screening interventions, including those in in rural and impoverished geographies.

This study has several strengths, including the use of high-quality population-based data from Unted States registries and a large sample size including counties across all U.S. census regions, further enhancing generalizability of findings. We were also able to assess the trends in breast cancer stage at diagnosis over the COVID-19 pandemic over time, which had not been previously assessed. Potential study limitations include the categorization of counties into dichotomous rural/urban categories, which may not capture nuanced differences across the rural to urban continuum. Additionally, we incorporated county-level data on demographic factors that were not specific to our study population (females between the ages of 20–74 years). Additionally, the SEER data warning for 2020 presented a challenge to our study design. To address this issue, we stratified our analysis by excluding 2020 data from our main analysis. Lastly, our analysis was ecological in nature and cannot account for individual-level factors that may have impacted breast cancer diagnosis rates at varying stages. Future studies utilizing individual-level data can shed light on socioeconomic, geographic, and environmental barriers to receiving timely breast cancer screening and can inform efforts to improve breast cancer screening and early detection rates in the wake of the COVID-19 pandemic.

## Conclusion

Taken together, results from this study suggest delays in breast cancer diagnosis experienced in persistently poor counties, particularly in rural counties and those with large Non-Hispanic Black populations. Importantly, LSBC rates did not differ by persistent poverty status after adjusting for recent poverty, suggesting the potential effectiveness of interventions targeting current socioeconomic barriers to mitigate breast cancer stage at diagnosis disparities. Rising rates of late-stage breast cancer in recent years were observed, with the most pronounced increases in persistently poor counties. These findings emphasize the importance of continued evaluation of trends in breast cancer diagnosis by stage, particularly in persistently poor, rural areas and in counties with large Non-Hispanic Black populations. Future studies should also evaluate patterns and trends in stage at diagnosis for other cancers for which screening improves cancer outcomes (i.e., colorectal, cervical, and lung cancers) and consider interventions like health insurance enrollment, establishing community health centers in remote areas, and creating tailored educational programs to promote screening. Continued surveillance at the local, state, and national level will inform future efforts to mitigate the impact of national emergencies on breast cancer screening and may elucidate barriers to screening within persistently impoverished areas, as well as among rural populations and racial and ethnic minorities.

## Supplementary Material

This is a list of supplementary files associated with this preprint. Click to download.

• SupplementalFigure1.docx

## Figures and Tables

**Table 1:** County-level characteristics by persistent poverty status from 2017–2021 (N = 1,079).

Characteristic	All Counties (N = 1,079)	Persistent Poverty (n = 156)	Non-persistent Poverty (n = 923)
**Rural/Urban Status, n (%)**			
Rural	639 (59.2)	127 (81.4)	512 (55.5)
Urban	440 (40.8)	29 (19.6)	411 (44.5)
**Census Region, n (%)**			
Northeast	105 (9.7)	1 (0.6)	104 (11.3)
South	592 (54.9)	138 (88.5)	454 (49.2)
Midwest	201 (18.6)	3 (1.9)	198 (21.5)
West	181 (16.8)	14 (9.0)	167 (18.1)
**% Non-Hispanic Black, mean (SD)**	9.42 (13.6)	21.14 (21.5)	7.45 (10.6)
**% Hispanic or Latino, mean (SD)**	15.12 (19.1)	18.20 (28.9)	14.60 (16.9)
**% Recent Poverty, mean (SD)**	16.01 (6.6)	25.99 (5.9)	14.32 (4.9)
**% Without Health Insurance, mean (SD)**	17.54 (8.7)	23.59 (9.7)	16.52 (8.1)
**% Received Bachelor’s Degree or Higher, mean (SD)**	20.77 (3.8)	14.41 (6.4)	21.84 (9.6)
**% Unemployment, mean (SD)**	3.76 (1.4)	4.55 (1.8)	3.62 (1.3)

**Table 2:** Mean late-stage BC incidence rates (per 100,000 person-years) in U.S. counties (N-1,079) from 2017–2021 (excluding 2020) according to persistent poverty status, overall and by rural/urban status and percentage Non-Hispanic Black population.

	All Counties		Persistent Poverty		Non-persistent Poverty		
	N	Mean Late-Stage Incidence Rate(SD)	n	Mean Late-Stage Incidence Rate(SD)	n	Mean Late-Stage Incidence Rate(SD)	*p* ^a^
Overall	1,079	54.03 (19.7)	156	58.98 (24.0)	923	53.20 (18.7)	0.005
**Rural/Urban Status**							
Rural	639	53.48 (23.0)	127	58.61 (24.8)	512	52.20 (22.3)	0.005
Urban	440	54.84 (13.4)	29	60.59 (20.4)	411	54.44 (12.7)	0.12
**Non-Hispanic Black**							
> 5%	443	56.64 (17.5)	85	61.67 (25.0)	358	55.40 (15.0)	0.03
≤ 5%	636	52.22 (20.9)	71	55.76 (22.6)	565	51.78 (20.6)	0.13

a p-values calculated from two-sample independent t-test comparing persistent poverty and non-persistent poverty counties.

**Table 3:** Late-stage breast cancer incidence rate difference (RD) estimates (per 100,000 person-years) between persistent poverty and non-persistent poverty counties in the US from 2017–2021 (excluding 2020), stratified by rural/urban status and percentage Non-Hispanic Black (N = 1079).

	Number Persistent Poverty/ Non-persistent Poverty Counties	Age-adjusted Rate Difference	Multivariable^a^ Rate Difference	p-value_int_^b^
**All Counties**	156/923	5.78 (2.5, 9.1)	2.74 (−1.6, 7.1)	N/A
**Rural/Urban Status**				
Rural	127/512	6.41 (2.0, 10.9)	2.30 (−3.9, 8.5)	0.94
Urban	29/411	6.16 (1.1, 11.2)	2.73 (−3.1, 8.6)	
**Non-Hispanic Black**				
> 5%	85/358	6.23 (2.1, 10.3)	−1.26 (−6.7, 4.2)	0.08
≤ 5%	71/565	3.98 (−1.2, 9.1)	7.23 (0.2, 14.2)	

a Multivariable model adjusted for recent poverty, rural-urban continuum code, % without health insurance, % unemployed, % Non-Hispanic Black, % Hispanic or Latino, and % with a bachelor’s degree or higher.

b p-value for interaction based on multivariable model.

## Data Availability

The data analyzed in this study were obtained from the National Cancer Institute’s Surveillance, Epidemiology, and End Results (SEER) Research Plus Limited-Field 22 database.
